# Association between arachidonate lipoxygenase 15,c.-292 C > T gene polymorphism and non-cystic fibrosis bronchiectasis in children: a pilot study on the effects on airway lipoxin A4 and disease phenotype

**DOI:** 10.1186/s13052-024-01654-5

**Published:** 2024-04-29

**Authors:** Mahitab Morsy Hussein, Eman Mahmoud Fouda, Yasmine Shehab, Enas Samir Nabih, Ahmed Mohamed Osman, Sally Raafat Ishak

**Affiliations:** 1 Pediatrics Department, Faculty of Medicine, Ain Shams University, Cairo, Egypt; 2 Medical Biochemistry and Molecular Biology Department, Faculty of Medicine, Ain Shams University, Cairo, Egypt; 3 Radiology Department, Faculty of Medicine, Ain Shams University, Cairo, Egypt

**Keywords:** ALOX-15 polymorphism, BAL, Lipoxin A4, Bronchiectasis

## Abstract

**Background:**

Persistent airway inflammation is a central feature of bronchiectasis. Arachidonate 15-lipoxygenase (ALOX-15) controls production of endogenous lipid mediators, including lipoxins that regulate airway inflammation. Mutations at various positions in ALOX-15 gene can influence airway disease development. We investigated association between *ALOX-15,c.-292 C > T *gene polymorphism and bronchiectasis unrelated to cystic fibrosis in Egyptian children. Also, lipoxin A4 (LXA4) level in bronchoalveolar lavage (BAL) was studied in relation to polymorphism genotypes and disease phenotypes determined by clinical, pulmonary functions, and radiological severity parameters.

**Methods:**

This was an exploratory study that included 60 participants. Thirty children with non-cystic fibrosis bronchiectasis (NCFB) were compared with 30 age and sex-matched controls. *ALOX-15,c.-292 C > T *polymorphism was genotyped using TaqMan-based Real-time PCR. LXA4 was measured in BAL using ELISA method.

**Results:**

There was no significant difference between patients and controls regarding *ALOX-15,c.-292 C > T* polymorphism genotypes and alleles (OR = 1.75; 95% CI (0.53–5.7), *P* = 0.35) (OR = 1; 95% CI (0.48-2), *p* = 1). BAL LXA4 level was significantly lower in patients, median (IQR) of 576.9 (147.6–1510) ng/ml compared to controls, median (IQR) of 1675 (536.8–2542) (*p* = 0.002). Patients with severe bronchiectasis had a significantly lower LXA4 level (*p* < 0.001). There were significant correlations with exacerbations frequency (*r*=-0.54, *p* = 0.002) and FEV1% predicted (*r* = 0.64, *p* = 0.001). Heterozygous *CT *genotype carriers showed higher LXA4 levels compared to other genotypes(*p* = 0.005).

**Conclusions:**

Low airway LXA4 in children with NCFB is associated with severe disease phenotype and lung function deterioration. *CT *genotype of *ALOX-15,c.-292 C > T *polymorphism might be a protective genetic factor against bronchiectasis development and/or progression due to enhanced LXA4 production.

**Supplementary Information:**

The online version contains supplementary material available at 10.1186/s13052-024-01654-5.

## Background

Bronchiectasis is a syndrome characterized by endobronchial suppuration, inflammation, and impaired mucus clearance with the end result of chronic microbial infection and progressive decline in lung function [1]. Clinically, it is defined by chronic or recurrent wet cough confirmed by evidence of bronchial dilation in high-resolution computed tomography (HRCT) [2]. While cystic fibrosis (CF) is a major etiology, bronchiectasis unrelated to CF is associated with various pathological conditions. Immunodeficiency disorders, severe infections, aspiration, primary ciliary dyskinesia, and airway anomalies represent the most common etiologies [1]. It is often classified as idiopathic if underlying cause is undetermined [3]. Burden of non-CF bronchiectasis (NCFB) is still unclear. A prevalence of 0.2–735 cases per 100,000 children is suggested [4]. Persistent airway inflammation is a central feature of bronchiectasis [5] in the presence or absence of bacterial infection [6]. Studies have characterized inflammatory exudate to include abundant neutrophilic infiltrate, elevated proinflammatory cytokines as interleukin (IL)-8, IL-6, IL-1β, tumor necrosis alpha (TNFα), and anti-microbial peptides as interferon gamma-induced protein − 10 (IP-10) and LL-37 [7]. Watt and colleagues [8] also found prolonged airway neutrophil survival in bronchiectasis. The elevated neutrophil count was strongly correlated with impaired lung function and bronchiectasis severity [9]. Dysregulated inflammatory response to repeated environmental insults has been proposed as host immunologic mechanism contributing to uncontrolled inflammation in pediatric NCFB. However, orchestrator for this is still unknown [10]. This highlighted the role of endogenous anti-inflammatory pathways in progression of NCFB. Failure of eicosanoid class switching with abnormal lipoxins production has been previously studied in the CF lung [11]. Lipoxin A4 (LXA4) belongs to a class of newly identified specialized pro-resolving lipid mediators (SPMs) playing a central role in resolution of inflammation as they inhibit neutrophils effector functions [12]. They are first eicosanoids to be expressed during process of class switching from prostaglandins and leukotrienes (LTB4), followed by resolvins and protectins [13]. LXA4 biosynthesis involves a multistep enzymatic process initiated by lipoxygenation of arachidonic acid by 15-lipoxygenase (ALOX-15) enzyme in macrophages and airway epithelial cells blocking LTB4 biosynthesis [14]. Thus, ALOX-15 pathway plays an important role in underlying pathogenesis of airway inflammation [15] Arachidonic acid 15-lipoxygenase (ALOX-15) gene, located on chromosome 17p13.3, controls transcriptional activity and hence, function of lipoxins [16] Eleven gene variations were detected. Functional polymorphisms in ALOX-15 gene could alter ALOX-15 enzyme activity and influence disease progression. A functional single nucleotide polymorphism (SNP) resulting from *C-to-T* substitution at position *c.-292* was found to increase gene transcription [17]. To the best of our knowledge, no previous studies investigated ALOX-15 gene polymorphisms and their influence on lipoxin production in airway of children with NCFB.

Our objective was to investigate the association between *ALOX-15,c.-292 C > T *gene polymorphism and NCFB in Egyptian children. Also, lipoxin A4 level measured in bronchoalveolar lavage (BAL) was studied in relation to polymorphism genotypes and disease phenotype determined by clinical, pulmonary functions, and radiological severity parameters.

## Methods

### Study design

This pilot study recruited 30 children with NCFB collected from the regular patients of Pediatric Pulmonology clinic, Children’s Hospital, Ain Shams University, Cairo, Egypt from December 2020 to December 2021. Patients were enrolled if they have clinical symptoms consistent with bronchiectasis confirmed radiographically using pediatric criteria for bronchial dilation in high-resolution computed tomography (HRCT) scan and a negative sweat test [18], age between 1 and 16 years and in a clinically stable state. Exclusion criteria included presence of acute exacerbation at time of enrollement defined as increased respiratory symptoms, mainly cough, increased sputum quantity or purulence for ≥ 3 days, dyspnea or hypoxia irrespective of the duration [19], confirmed cystic fibrosis (CF) diagnosis, underlying chronic inflammatory conditions, known or suspected chromosomal abnormality, on antibiotics or anti-inflammatory drugs including inhaled and systemic steroids four weeks before the study or immunosuppressive therapy. They were compared with 30 age and sex-matched previously healthy children referred for bronchoscopy in view of suspected foreign body aspiration. Only those with normal airway anatomy, and normal microbiology BAL results were included.

### Ethical considerations

#### Informed consent

was obtained from participants or their legal guardians before enrollment. This study was performed in line with principles of the Declaration of Helsinki 1975. Approval was granted by Research Ethics Committee of human experimentation, Faculty of Medicine, Ain shams university (FMASU MD 270/2020).

### Data collection

All patients were subjected to detailed history taking, laying stress on demographics, disease duration, symptoms suggestive of exacerbation, and frequency of hospitalization due to exacerbations in last 12 months. Vital data and anthropometric parameters, including weight, height, and body mass index (BMI), were recorded and plotted on centiles. Pulse oximetry was used to monitor oxygen saturation.

Routine inflammatory markers as complete blood count (CBC) and C-reactive protein (CRP) were performed at enrollment. Patients and controls with abnormal results were not included.

### Non-CF bronchiectasis severity assessment tools

1. Pulmonary function tests.

Forced spirometry was performed for enrolled subjects using JAEGER apparatus, care fusion, Germany, 2011. Standard methodology for acceptability and repeatability of spirometry was followed according to combined American thoracic society / European respiratory society guidelines [19]. The following parameters were obtained: forced vital capacity (FVC), forced expiratory volume in first second (FEV1), ratio between FEV1 and FVC (FEV1/FVC), and maximal mid-expiratory flow rate during 25–75% of expiration (MMEF25-75) and results were expressed as percentage (%) of predicted values based on age, sex, ethnicity, weight, and height. Spirometry was interpreted as normal if FEV1 and FVC % predicted were ≥ 80% and FEV1/FVC ratio > 80. Severity of obstructive ventilatory defect was graded based on FEV1% predicted values as follows: mild if > 70%, moderate if < 70 to 50%, severe if < 50 to 30% predicted [20].

2. Radiological evaluation using Modified Bhalla score and quantitative lung analysis.

High-resolution computed tomography (HRCT) scan was performed for all enrolled patients using a 64-slice CT machine (Optima CT, GE “general electric”, USA). No specific preparations were required. Only in non-cooperative children, general anesthesia was needed to obtain scanning during suspended inspiration. In older children, scanning was taken during full inspiration. Patients were scanned in supine position with arms above the head. Image acquisition was at 1.25 mm thickness, 0.625 mm intervals using 512 × 512 matrix, tube speed 35 mm/rotation with 0.5s rotation time. The KVp and mAs were used as low as possible to limit radiation dose. Image analysis was done by an experienced radiologist blinded to patient’s condition. The following items were recorded: distribution and severity of bronchiectasis, peribronchial thickening, and severity, presence of mucus plugging, sacculations, bullae/emphysema, and consolidation/collapse. Modified Bhalla score [21] was calculated to assess radiological disease severity. Total score range from 0 to 37.

Post-processing, using Fuji 3D synapse automated software, a quantitative analysis based on CT image (CT volumetry) was performed, where volume of the diseased areas was calculated as a percentage of the total lung volume. This was referred to as percentage of lung volume affected by bronchiectasis [22].

3. Pediatric bronchiectasis severity index.

A pediatric-specific score was adapted as an assessment tool of disease severity in studied subjects. It uses a combination of clinical (age, nutritional status, exacerbations, hospital admissions over a year period, and patient compliance), radiological and microbiological features. Depending on calculated score, disease was graded as mild if 0–4, moderate if 5–8, and severe if ≥ 9 [23]. Chronic airway colonization was determined if a pathogenic microorganism was identified in cultures from BAL and/or induced sputum samples on at least 2 occasions, 3 months apart in the previous 12 months [24].

4. Modified medical research council (mMRC) dyspnea scale.

Degree of perceived breathlessness and respiratory dysfunction in daily living activities of enrolled patients was evaluated using mMRC scale. It is a self-reported five-statement questionnaire, ranging from grade 0 (dyspnea only on strenuous exercise) to grade 4 (dyspnea on dressing/undressing) [25].

### Laboratory work-up

1. Measurement of Lipoxin A4 level in bronchoalveolar lavage fluid (BALF).

All enrolled patients and controls underwent bronchoscopy with bronchoalveolar lavage (BALF) collection according to European respiratory society guidelines [26]. Bronchoscopy was done under general anesthesia through a laryngeal mask. Standardized protocol for BALF collection under aseptic conditions was followed by wedging in right middle lobe bronchus and lingula, then sterile normal saline (1-2 ml/kg) was instilled and suctioned immediately. Collected samples were transported in sterile containers to laboratory where total and differential cytology was immediately determined, microbiological cultures were performed and a sample was stored at -20◦C for further use. BAL samples were centrifuged for 20 min at 1000xg. The supernatants were collected for measurement of lipoxin A4 concentration using a human lipoxin A4 ELISA kit (E0612Hu, Bioassay Technology Laboratory, China). LXA4 in samples and standards competed with that is coated to wells for the Biotinylated Detection antibody specific to LXA4. After a washing step, an Avidin-Horseradish peroxidase (HRP) conjugate was added and incubated followed by addition of a TMB substrate solution. The reaction was terminated after 10 min by the stop solution. The optical density was measured at 450 nm and concentration of LXA4 was calculated using a standard curve with an intra- & inter-assay CV of 5.6 & 7.7 respectively, a range of 5-2000 ng/ml and sensitivity of 2.47ng/l.

2. Genotyping of *ALOX-15,c.-292 C > T* (rs2072510) single nucleotide polymorphism (SNP) using TaqMan-based Real-time PCR.

Venous blood samples were collected from all participants under aseptic conditions in EDTA-containing tubes and stored at -20◦C. DNA was extracted from plasma samples using QIAamp DNA blood kits, catalog no: 51,104 (Qiagen, Hilden, Germany). DNA concentration of 50 ug/ml was determined and DNA purity was detected by determination of A_260_/A_280_ ratio. All patients and controls were genotyped for *ALOX-15,c.-292 C > T* SNP using Applied Biosystems TaqMan SNP genotyping assays ”ALOX-15 C/T rs2072510, catalog no:4,351,379 (ThermoFisher Scientific, Germany) and PCR TaqMan Genotyping Master Mix kit, cat no:4,371,353 (ThermoFisher, Germany). The thermal cycling protocol was optimized as follows: 95◦C for 10 min for AmpliTaq Gold, UP enzyme activation, followed by denaturation step at 95◦C for 15 s and annealing/extension at 60◦C for 1 min for 40 cycles. The qPCR was performed on Applied Biosystems PCR instrument (ThermoFisher Scientific, Germany).

### Statistical analysis

There were no previous data to inform a power calculation, so sample size in this pilot study is opportunistic based on availability of samples. 60 participants is a conservative estimate to detect a statistically significant result. Statistical package for social science, version 23.0 (SPSS Inc., Chicago, Illinois, USA) was used for data management and statistical analysis. Quantitative variables were presented as mean, standard deviation (SD), and ranges when parametric, median, and interquartile range (IQR) when non-parametric. Categorical variables were presented as numbers (n) and percentages (%). Chi-square test was used for comparison between 2 groups regarding qualitative data. For comparison between 2 groups with quantitative variables, independent t-test (parametric) and Mann-Whitney test (non-parametric) were used. Kruskall-Wallis test was used for comparison between more than 2 quantitative variables (non-parametric). Alleles frequency was calculated using gene counting method, Chi-square was used to test the difference between groups as regards genotypes and alleles and to prove Hardy-Weinberg equilibrium. Association of Genetic polymorphism with bronchiectasis was assessed by exact logistic regression model, odds ratio (OR), and 95% confidence interval (CI) were calculated. Spearman correlation coefficients were used for correlation analysis. Confidence interval was set at 95%, margin of error accepted was set at 5%. P-value was considered significant if < 0.05.

## Results

This study included 30 pediatric patients with non-CF bronchiectasis of post-infectious or idiopathic etiology, they were 18 females (60%) and 12 males (40%), and their ages ranged from 3 to 15 years old with mean (SD) of 8.87 (3.6) years. They were well-matched with controls regarding age and sex.

BAL Lipoxin A4 levels and distribution of *ALOX-15,c.-292 C > T* gene polymorphism genotypes and alleles among patients and controls are shown in Table 1.

**Table 1:** Demographics, BAL lipoxin A4 level, ALOX-15, c.292 C> T polymorphism genotypes and alleles distribution in patients and controls

	Controls	Patients	OR (95% CI)		P-value
	*N* = 30	*N* = 30			
**Gender**					
Females Males	16 (53.3%) 14 (46.7%)	18 (60.0%) 12 (40.0)	-	0.27*	0.602
**Age (years)** ^**a**^	7.43 ± 2.94 3– 14	8.87 ± 3.60 3– 15	-	-1.68•	0.097
**BAL Lipoxin A4 (ng/ml)** ^**b**^	1675 (536.8– 2542) 370.6– 3876	576.9 (147.6– 1501) 68.8– 2352	-	-3.13†	0.002
**ALOX-15 gene polymorphism**					
Genotypes, n (%)					
CC CT TT	14 (46.7%) 8 (26.7%) 8 (26.7%)	12 (40.0%) 12 (40.0%) 6 (20.0%)	Referent* 1.75 (0.53-5.70) 0.87 (0.23– 3.24)		- 0.353 0.842
Alleles frequency, n (%)					
C T	36 (60.0%) 24 (40.0%)	36 (60.0%) 24 (40.0%)	Referent* 1 (0.48 to 2.07)		- 1

a: median±SD, range; b: median (IQR), range; *: chi-square test; •: Independent t-test; †:Mann-Whitney test

OR: odds ratio; CI: confidence interval; BAL: bronchoalveolar lavage, ALOX-15: arachidonate 15-lipoxygenase

Patients with NCFB showed a significantly lower level of LXA4, median (IQR) of 576.9 (147.6–1510) ng/ml, when compared to controls (*p* = 0.002) (Figure S1). However, this difference was insignificant among *CT* genotype carriers, median (IQR) 1519 (918.1–2073) (*p* = 0.35) (Figure S2). Lipoxin A4 level was significantly higher among *CT* genotype compared with *CC*, median (IQR) 546.5 (155.75–971) and *TT *genotypes, median (IQR) 140.6 (132.7–256) (*p* = 0.005) (Table 2).

**Table 2:** Association between ALOX-15 genotypes and BAL lipoxin A4 level, pediatric bronchiectasis severity score, chronic airway colonization, and nutritional status in patients

	ALOX-15 Genotypes, *n*=30			t	P-value
	CC	CT	TT		
	*N* = 12	*N* = 12	*N* = 6		
**BAL Lipoxin A4 level** ^**b**^ **(ng/ml)**	546.5 (155.75– 971.05) 68.8– 2045	1519 (918.1– 2073) 89– 2352	140.6 (132.7– 256) 121.5– 582	10.64§	0.005
**BMI Z-score** Low Normal	4 (33.3%) 8 (66.7%)	6 (50.0%) 6 (50.0%)	5 (83.3%) 1 (16.7%)	4*	0.135
**Chronic airway colonization** * No* * Yes*	4 (33.3%) 8 (66.7%)	3 (25.0%) 9 (75.0%)	1 (16.7%) 5 (83.3%)	0.59*	0.742
**Pediatric bronchiectasis severity index** Mild Moderate Severe	2 (16.7%) 8 (66.7%) 2 (16.7%)	6 (50.0%) 5 (41.7%) 1 (8.3%)	0 (0.0%) 4 (66.7%) 2 (33.3%)	6.79*	0.147
**PFT affection and severity grading** Normal Mild Moderate Severe	2 (16.7%) 3 (25.0%) 6 (50.0%) 1 (8.3%)	1 (8.3%) 3 (25.0%) 5 (41.7%) 3 (25.0%)	1 (16.7%) 1 (16.7%) 3 (50.0%) 1 (16.7%)	1.62*	0.951
**Modified Bhalla score** ^**b**^	9 (4.5 − 13.5) 0– 20	9.5 (5.5 − 14) 0– 20	11.5 (8 − 15) 5– 15	0.513§	0.77

b: median (IQR), range; §:Kruskal Wallis test; *:Chi-Square test

ALOX-15: arachidonate 15-lipoxygenase; BAL: bronchoalveolar lavage; BMI: body mass index; PFT: pulmonary function test; C: cytosine; T: thymine

Clinical and disease severity characteristics of studied patients are illustrated in Table 3. Bronchiectasis was moderate in 60%, mild in 23%, and sever in 16.7% of patients. 4 patients (14.8%) had normal spirometry results. Obstructive ventilatory defect was graded as mild in 25.9%, moderate in 40.7%, and severe in 18.5% of patients. Neutrophils were predominant cell type in BAL (76.7%). Chronic airway colonization was present in 70% of patients with haemophilus influenzae being most commonly isolated organism.

**Table 3:** Patients’ clinical characteristics and disease severity assessment

Variable	Patients
	*N*=30
**Duration of illness (years)** ^b^	8 (5 - 9) 2– 14
**Frequency of admissions due to exacerbations during last year** ^b^	1.5 (1– 2) 1– 4
**Pediatric Bronchiectasis severity index, n (%)** Mild Moderate Severe	7 (23.3%) 18 (60%) 5 (16.7%)
**mMRC dyspnea scale, n (%)** Grade 0 Grade 1 Grade 2 Grade 3	13 (43.3%) 12 (40%) 4 (13.3%) 1 (3.3%)
**BMI for age (kg/m2)** ^**a**^	14.87 ± 1.99 10.8– 18.6
**BMI Z-score interpretation** Normal Low	15 (50%) 15 (50%)
**Modified Bhalla score** ^**b**^	9.5 (5– 14) 5– 20
**Percentage of Lung volume affected by bronchiectasis in HRCT (%)** ^**b**^	10 (7– 35) 5– 70
**Spirometry results** ^**a**^ FVC (% predicted)	80.74 ± 19.46 22.6– 107
FEV1 (% predicted)	70.94 ± 19.72 25.8– 109
FEV1/FVC	91.36 ± 16.92 56– 115
MMEF (% predicted)	64.32 ± 18.20 34– 92
**PFT interpretation and severity grading (** ***n*** **=27)** Normal Mild Moderate Severe	4 (14.8%) 7 (25.9%) 11 (40.7%) 5 (18.5%)
**Total BAL cell count (c/mm3)** ^**b**^	750 (275– 1200) 166– 16,000
**Predominant BAL cell type** Neutrophils Lymphocytes	23 (76.7%) 7 (23.3%)
**Chronic airway infection** No Yes	9 (30%) 21 (70%)
**Type of organism, n(%)** H. influenzae MRSA Pseudomonas aeruginosa Strept. Pneumoniae	11 (36.7%) 4 (13.3%) 4 (13.3%) 2 (6.7%)

a: mean±SD, range; b: median (IQR), range

PFT: pulmonary function test; BAL: bronchoalveolar lavage; MRSA: methicillin resistant staphylococcus aureus, Strept. Pneumoniae: streptococcus pneumoniae; FVC: forced vital capacity; FEV1: forced expiratory volume in first second; MMEF: mid maximal expiratory flow rate; BMI: body mass index; mMRC: modified medical research council dyspnea scale

Table 4 shows that LXA4 was significantly lower in patients with severe disease with median (IQR) of 132.7 (89-138.9) compared to those with mild and moderate disease (*p* < 0.001). Patients with chronic airway infection had lower levels of LXA4, however, this was statistically non-significant. Patients with normal lung functions showed a significantly higher level of LXA4 (*p* = 0.04).

**Table 4:** Association between BAL lipoxin A4 level and pediatric Bronchiectasis severity score, mMRC dyspnea scale, pulmonary functions, and chronic airway colonization in patients

	BAL lipoxin A4 level (*n*=30)		P-value
	Median (IQR)		
**Gender** Female Male	564.3 (149.4-1408) 576.9 (276.1-1554.5)	0.38¶	0.70
**Pediatric Bronchiectasis severity index** Mild Moderate Severe	1708 (1477.5– 2186) 576.9(256– 1408) 132.7 (89– 138.9)	15.52§	0.00
**PFT affection and severity grading** Normal Mild Moderate Severe	1113.95 (578.9– 2336.2) 1408 (476– 1572) 1844 (256– 2302) 576.9 (149.4– 1241)	8.23§	0.04
**mMRC dyspnea scale** Grade 0 Grade 1 Grade 2 Grade 3	599.1 (410-1537) 319 (131.1-951.2) 995 (362.1-1413) 1844	3.97§	0.26
**Chronic airway colonization** Not colonized Colonized	585.45(274.45– 1690.5) 570.75(149.4– 1418)	0.23¶	0.81

Mann-Whitney test; §: Kruskal Wallis test BAL: bronchoalveolar lavage; IQR: interquartile range; PFT: pulmonary function test; mMRC: modified medical research council

**Fig. 1:**
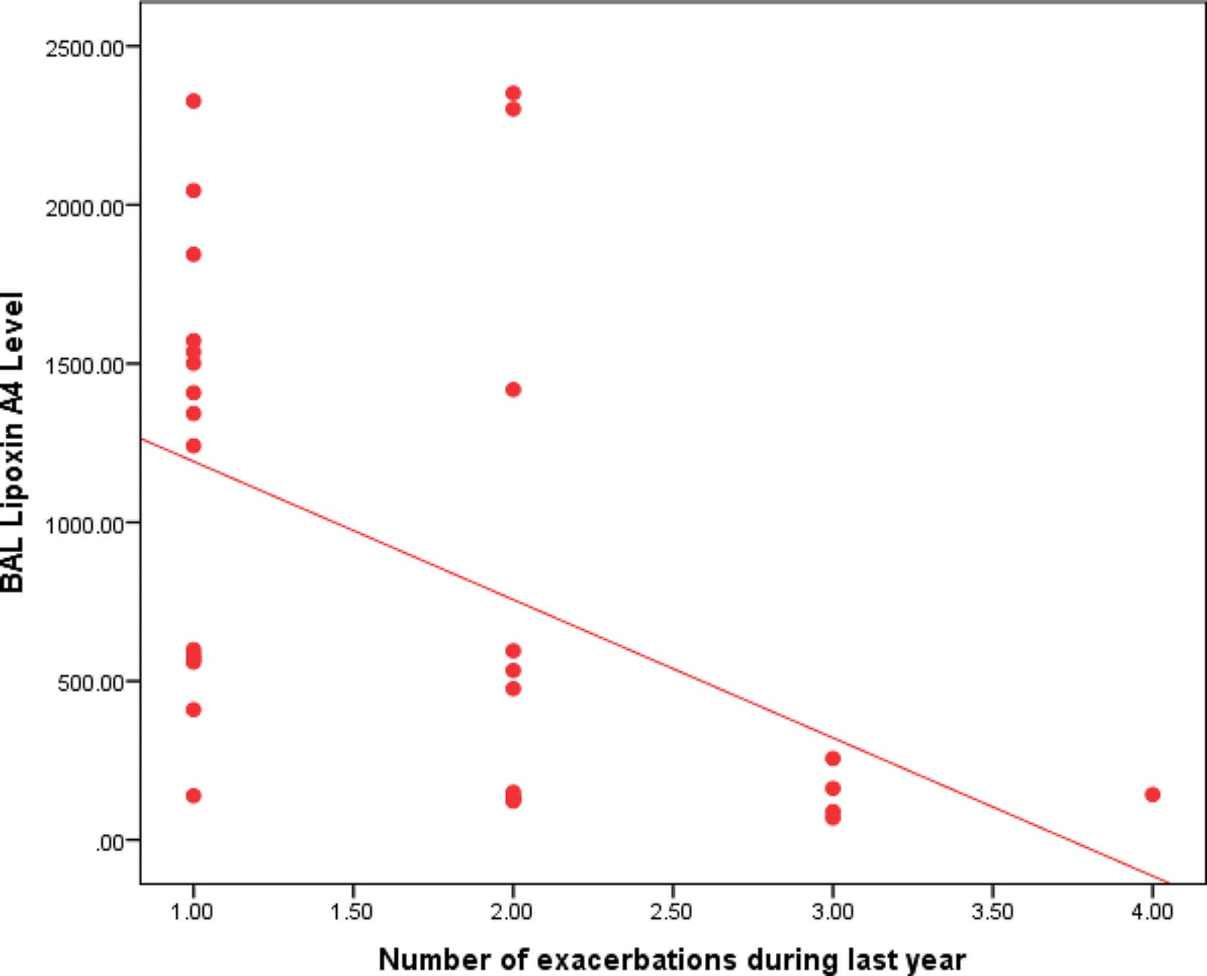
Correlation between BAL lipoxin A4 and frequency of admissions due to exacerbations over past year (*r*= -0.54, *p*=0.002) r: Spearman correlation coefficient

There was significant correlation with FEV1% predicted (*r* = 0.64,*p* = 0.001) (Figure S3) and frequency of hospitalization due to exacerbations over past 12 months (*r*=-0.54, *p* = 0.002) (Fig. 1).

Comparison between *ALOX-15,c.-292 C > T *polymorphysim genotypes showed no significant difference as regards disease severity index (*p* = 0.14), abnormal pulmonary function results (*p* = 0.95), radiological severity score, chronic airway infection (*p* = 0.74) and nutritional status (*p* = 0.13) among studied subjects (Table 2).

Logistic regression analysis detected no significant association between *ALOX-15,c.-292 C > T* genetic polymorphism and non-CF bronchiectasis in studied subjects (Table 5).

**Table 5:** Exact logistic regression model showing the association between ALOX-15 genotypes, alleles, and non-CF bronchiectasis

	Controls	Patients	OR (95% CI)	P-value
	*N* = 30	*N* = 30		
ALOX-15 polymorphism Genotypes	46.7 / 26.7 / 26.7	40.0 / 40.0 / 20.0	1.3 (0.37- 4.58)	0.66
Alleles	60.0/40.0	60.0/40.0	1 (0.40-2.44)	1

OR: Odds ratio; 95% CI: 95% confidence interval; ALOX: arachidonate 15-lipoxygenase

## Discussion

In this study, we attempted to scrutinize the role of ALOX-15 gene polymorphism and LXA4 in pediatric NCFB, an area hitherto not extensively explored. NCFB is multifactorial, predisposed by genetic factors with a poorly understood pathogenesis [3]. Considerable efforts were made to understand underlying exaggerated and/or dysregulated inflammation in response to challenges from respiratory pathogens. Data from CF or chronic obstructive pulmonary diseases (COPD) were extrapolated, although they are distinctly different entities [11]. Though previously studied in relation to asthma [27], COPD [28], CF [29], and other systemic diseases [30], role of LXA4 in NCFB in children has not yet been elucidated. This research presented an insight into the intricate relationship between ALOX-15 gene, LXA4, and pediatric NCFB. It pointed out that low LXA4 level in the airway of children with NCFB was associated with clinical disease severity in the form of reduced lung functions and increased exacerbations frequency. *ALOX-15,c.-292 C > T* SNP was functional and increased LXA4 level among heterozygous CT carriers. However, its association with NCFB occurrence and its severity was not detected. In our study, BAL lipoxin A4 level was significantly lower in children with NCFB compared to controls (*p* = 0.002). Also, it was significantly lower in patients with severe disease according to pediatric bronchiectasis severity score (*p* = 0) and pulmonary function parameters (*p* = 0.04). There was no significant difference between those with and without chronic airway infection (*p* = 0.81). This was in agreement with Bedi et al. [31] who studied LXA4 in adult subjects with bronchiectasis. Celik et al. [27] and Balode et al. [28] reported same findings regarding LXA4 in relation to asthma and COPD, respectively. Ringholz et al. [12] and Urbach et al. [29] studied airway LXA4 in children with CF and it was significantly reduced compared to controls, even in the absence of infection. The documented correlation of low LXA4 levels with disease severity underscores the potential significance of resolving lipid mediators in modulating inflammation and disease progression. LXA4 contributes to active resolution of inflammation by inhibiting LTB4-induced neutrophils recruitment, antagonizing the effect of pro-inflammatory mediators as IL-8, myeloperoxidase, and reactive oxygen species limiting tissue injury. Also, it promotes neutrophil apoptosis in the inflammatory site through enhancement of phagocytosis by macrophages [32]. Karp et al. [33] reported that mice treated with LXA4 and challenged with pseudomonas aeruginosa contained the infection effectively. The use of lipoxin gained attention recently in many diseases associated with an excessive inflammatory response as its deficiency has been associated with a wide range of pathologies. Impaired resolution of inflammation may be enhanced by genetic polymorphisms affecting SPM biosynthesis [34].

The influence of *ALOX-15,c.-292 C > T* gene polymorphism on NCFB in children was studied. There was no significant difference between patients and controls as regards distribution of *ALOX-15,c.-292 C > T *genotypes. *T* allele frequency was equal among patients and controls [OR = 1, 95% CI (0.48-2), *p* = 1]. No significant association was detected between *ALOX-15,c.-292 C > T* polymorphism variants, and occurrence of NCFB which might pose questions about the genetic determinant’s role in disease pathogenesis. Few studies evaluated the effect of ALOX-15 gene polymorphism on LXA4 levels [35]. Also, its anti-inflammatory role was studied in animals [36]. Serhan et al. [37] showed that overexpression of arachidonate 15-LO in transgenic rabbits led to enhanced endogenous anti-inflammation. LXA4 level was significantly higher among heterozygous *CT *carriers compared to homozygous *CC* carriers (*p* = 0.005). Wittwer et al. [38] reported that polymorphism in ALOX-15 gene promotor region that involves *C-to -T* substitution at position *c.-292* leads to higher ALOX-15 enzyme transcription in macrophages from heterozygous *CT* carriers compared to homozygous *CC* carriers by creating a new binding site for transcription factor SPI1 resulting in a significant increase in arachidonic acid pathway metabolites. Thus, possible enhancement of endogenous anti-inflammation due to increase in pro-resolving mediators, mainly lipoxins, can be postulated. In conclusion, offering promising insights into the interplay between genetics, lipid mediators, and pediatric NCFB, this pilot study presents a foundation for further extensive investigations to decipher the intricate mechanisms underlying bronchiectasis development and progression. Also, our findings provide a window for ALOX-15 pathway as a potential area of research regarding failure of resolution of inflammation in pediatric NCFB. small sample size, single-center approach, and studying a single molecule from arachidonic acid pathway are the limitations of the present study. Other pro-inflammatory molecules as leukotrienes are also controlled by ALOX-15 enzyme. The multifactorial nature of bronchiectasis requires more comprehensive investigation encompassing broader genetic variations and gene-environment interactions [39]. Further research in this domain should consider expanding sample size, incorporating multi-omics approaches and exploring gene-enviroment interactions to unravel the complexity of NCFB pathophysiology comprehensively.

## Conclusions

Low airway LXA4 in children with NCFB is associated with severe disease phenotype and lung function deterioration. *CT* genotype of *ALOX-15,c.-292 C > T *polymorphism might be a protective genetic factor against bronchiectasis development and/or progression due to enhanced LXA4 production.

### Electronic supplementary material

Below is the link to the electronic supplementary material.

Supplementary Material 1

## Declarations

### Data availablity

All data generated or analyzed during this study are included in this published article or its supplementary information file.

## Abbreviations

HRCT: High resolution computed tomography
CF: Cystic fibrosis
NCFB: Noncystic fibrosis bronchiectasis
IL: Interlukin
TNFα: Tumor necrosis factor-alpha
Ip-10: interferon gamma-induced protein − 10
Alox-15: Arachidonic acid 15 lipoxygenase
SNP: Single nucleotide polymorphism
LXA4: Lipoxin A4
BALF: Bronchoalveolar lavage fluid
BMI: Body mass index
CBC: complete blood count
CRP: C reactive protein
FVC: Forced vital capacity
FEV1: Forced espiratory volume in first second
MMEF25-75: Maximal mid expiratory flow rate during 25–75% of expiration
mMRC: modified medical research council
