# Supplementary material for: Association between arachidonate lipoxygenase 15,c.-292 C > T gene polymorphism and non-cystic fibrosis bronchiectasis in children: a pilot study on the effects on airway lipoxin A4 and disease phenotype
Source: Ital J Pediatr. 2024 Apr 29;50:90. doi: 10.1186/s13052-024-01654-5 (PMC11059722; doi:10.1186/s13052-024-01654-5)
Supplement: Supplementary file 1 — Supplementary Material 1 [file 13052_2024_1654_MOESM1_ESM.docx]

**Supplementary file**

**Figure S1** Comparison between BAL Lipoxin A4 level in patients and controls


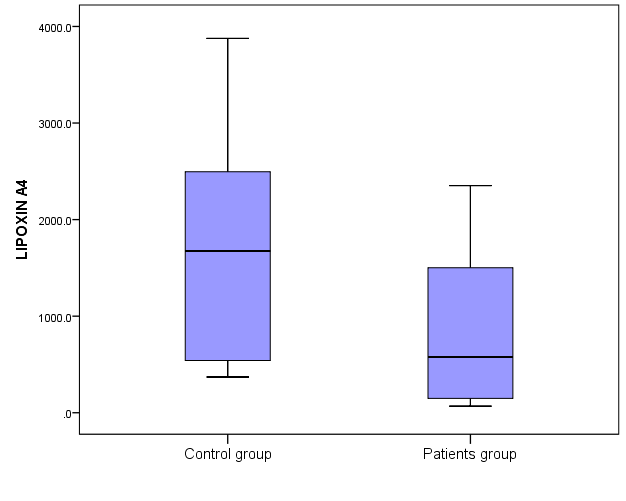


**Figure S2** Comparison between patients and controls as regards BAL lipoxin A4 among ALOX-15 polymorphism genotypes

**
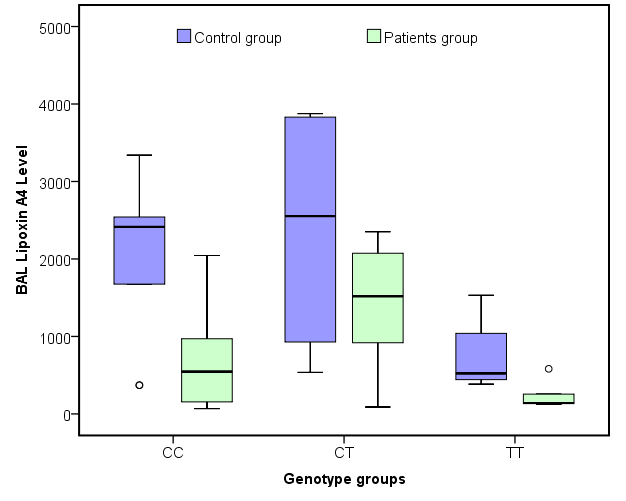
**


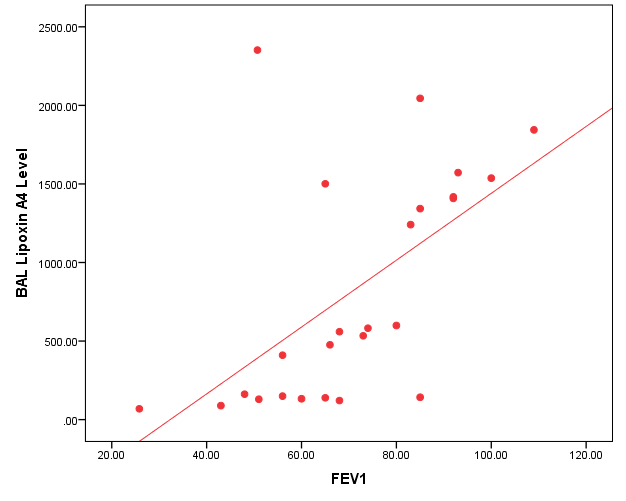


**Figure S3 Correlation between BAL lipoxin A4 and FEV1 % predicted (r=0.64, p=0.001)**

**r: Spearman correlation coefficient**
